# Nanoceria Coated with Maltodextrin or Chitosan: Effects on Key Genes of Oxidative Metabolism, Proliferation, and Autophagy in Human Embryonic Lung Fibroblasts

**DOI:** 10.3390/molecules30153078

**Published:** 2025-07-23

**Authors:** Elena V. Proskurnina, Madina M. Sozarukova, Elizaveta S. Ershova, Ekaterina A. Savinova, Larisa V. Kameneva, Natalia N. Veiko, Vladimir P. Saprykin, Khamzat K. Vyshegurov, Vladimir K. Ivanov, Svetlana V. Kostyuk

**Affiliations:** 1 Kurnakov Institute of General and Inorganic Chemistry of the Russian Academy of Sciences, Moscow 119071, Russia; s_madinam@bk.ru (M.M.S.); van@igic.ras.ru (V.K.I.); svet-vk@yandex.ru (S.V.K.); 2 Faculty of Chemistry, Biology and Biotechnology, North Ossetian State University Named After Kosta Levanovich Khetagurov, Vladikavkaz 362025, Russia; 3 State Scientific Center of the Russian Federation “Russian Scientific Center of Surgery Named After Academician B.V. Petrovsky”, Institute of Longevity with a Clinic of Rehabilitation and Preventive Medicine, Moscow 119435, Russia; es-ershova@rambler.ru (E.S.E.); satelit32006@yandex.ru (N.N.V.); 4 Department of Biology and General Genetics, Institute of Medicine, Patrice Lumumba Peoples’ Friendship University of Russia, Moscow 117198, Russia; savinova.ekaterina96@yandex.ru; 5 Research Centre for Medical Genetics, Moscow 115522, Russia; larisa.kameneva@yandex.ru; 6 Faculty of Biotechnology and Fisheries, K.G. Razumovsky Moscow State University of Technologies and Management, Moscow 109004, Russia; v_p_s@mail.ru; 7 Department of Faculty Therapy of the Medical Institute, Ingush State University, Magas 386001, Russia; hem1967@mail.ru

**Keywords:** nanoceria, maltodextrin, chitosan, cytotoxicity, genotoxicity, oxidative metabolism, proliferation, autophagy

## Abstract

Nanoceria is a multifaceted enzyme-like catalyst of ROS-mediated (reactive oxygen species) reactions, which results in its multiple biomedical applications. Biodegradable polysaccharide coatings improve biocompatibility, while the effects of these coatings on the ROS-related activity of nanoceria in cells need thorough studies. Here, we used human embryonic lung fibroblasts to study the effects of maltodextrin and chitosan coatings on cellular oxidative metabolism of nanoceria by examining cell viability, mitochondrial potential, accumulation of nanoparticles in cells, intracellular ROS, expression of NOX4 (NADPH oxidase 4), NRF2 (nuclear factor erythroid 2-related factor 2), NF-κB (nuclear factor kappa-light-chain-enhancer of activated B cells), and STAT3 (signal transducer and activator of transcription 3) proteins as well as the expression of biomarkers of DNA damage/repair, cell proliferation, and autophagy. Both types of polysaccharide-coated nanoceria were non-toxic up to millimolar concentrations. For maltodextrin-coated nano-CeO_2_, in contrast to bare nanoparticles, there was no oxidative DNA damage/repair with moderate activation of NOX4 expression. Like bare nanoceria, maltodextrin-coated nanoparticles demonstrate the proliferative impact and do not activate autophagy. However, maltodextrin-coated nanoparticles have an activating impact on mitochondrial potential and the NF-κB pathway. Chitosan-coated nanoceria causes short-term intracellular oxidative stress, activation of the expression of NOX4, STAT3, and NRF2, oxidative DNA damage, and double-strand breaks accompanied by activation of DNA repair systems. In contrast to maltodextrin-coated nanoparticles, chitosan-coated nanoceria inhibits the NF-κB pathway and activates autophagy. These findings would be useful in the development of advanced nanoceria-based pharmaceuticals and contribute to the understanding of the biochemical properties of nanoceria as a modulator of ROS-dependent signaling pathways.

## 1. Introduction

Nanoscale cerium dioxide (nanoceria) is a multifaceted enzyme-like catalyst of reactive oxygen species (ROS) reactions. The nanozyme properties of nanoceria depend on ROS concentration [1]. Nanoceria can act as superoxide dismutase [2], peroxidase [3], catalase [4], oxidase [5,6], phosphatase [7], photolyase [8], phospholipase [9], and nuclease [10]. Since reactive oxygen species are key participants in almost all vital processes in the body, nanoceria has great potential for applications in biomedicine through its excellent antioxidant, anti-inflammatory, and antibacterial properties [11,12]. Interestingly, nanoceria exhibits cytoprotective effects on normal cells, while it acts as a cytotoxic pro-oxidant in cancer cells with acidic pH [13]. Nano-CeO_2_ catalyzes production of molecular oxygen, which reduces hypoxia and enhances the therapeutic effects of photodynamic, photothermal, and radiation therapy as well as chemotherapy [13,14]. Due to neuroprotective, angiogenic, and regenerative features, nanoceria can be used in protecting the central nervous system [15,16] and the retina [17], and in tissue engineering for wound healing [18]. However, further research is needed into the long-term effects and the impact on the body’s biochemistry and genes.

Maltodextrin, which is a product of the partial hydrolysis of starch, can serve as a hydrophilic carrier for encapsulation of active pharmaceutical ingredients [19], nanosystems, proteins [20,21,22], and metal nanoparticles [23]. Maltodextrin is proposed as an effective biocompatible nanocarrier for intracellular drug delivery [24], for encapsulation of the unstable enzyme human tyrosine hydroxylase for delivery to neuronal cells and tissue [25], for bioavailable encapsulation of saffron extracts [26], proteins [21], components of green tea [27], spiramycin [28], and resveratrol [29], and in the development of intranasal vaccines [30]. Maltodextrin nanosponges can be prepared based on the principles of “green” chemistry [31]. Pure maltodextrin nanoparticles were not cytotoxic for A549 cells [20]. Biodegradability of acetylated or chitosan-linked maltodextrin can be regulated with pH [29,32]. A folic acid–maltodextrin polymer was used to modify Fe_3_O_4_–graphene oxide complex nanoparticles carrying doxorubicin [33]. The combination of maltodextrin with vitamin E significantly improved the stability of nano-emulsions [34]. A pH-sensitive nano-antibacterial system used a bacteria-specific maltodextrin transport pathway [35]. Coating rifampicin with maltodextrin allows it to penetrate dormant *S. aureus*, resulting in their reactivation and restoration of sensitivity to rifampicin, which opens up prospects for the treatment of persistent intracellular bacterial infections [36]. Despite many studies on maltodextrin coatings, there are no or very few articles devoted to maltodextrin-coated nanoceria.

On the contrary, there are many articles on chitosan-coated nanoceria. Chitosan is a biocompatible, non-toxic, and renewable natural polysaccharide that reacts with Ce(IV) to form a physiologically active chitosan-Ce(IV) complex [37]. A chitosan–nanoceria nanocomposite is an effective biocompatible nanoplatform for enzyme immobilization and sensor development [38,39]. Hybrid chitosan–nanoceria particles exhibit powerful antibacterial effects [40]. Cerium oxide nanoparticles loaded on chitosan show enhanced regenerative ability and biocompatibility for wound healing applications [41]. Yu et al. developed water-soluble chitosan nanoparticles with cerium oxide as eyedrops with antioxidant properties for the treatment of dry-eye syndrome [42]. The incorporation of cerium oxide nanoparticles enhanced the antibacterial activity of chitosan-based films against *E. coli* and *S. aureus* [43]. Chitosan nano-cocktails loaded with cerium oxide nanoparticles as an active drug and iron oxide nanoparticles as an imaging agent showed anti-inflammatory and anti-fibrotic efficacy in liver inflammation in mice [44]. A cerium oxide–chitosan nanocomposite loaded with ciprofloxacin exhibited enhanced antibacterial activity with hemocompatibility and no hepatotoxicity [45]. Sathiyaseelan et al. prepared and characterized 5-fluorouracil-modified chitosan nanoparticles with cerium oxide for enhanced anticancer activity in hepatocellular carcinoma [46]. Chitosan cryogels with nanoceria and tannic acid have excellent tissue adhesion, blood cell coagulation and hemostasis, and anti-infective and cell recruitment functions, which allow them to be used as wound dressings [47]. The combination of cerium oxide and peroxide in chitosan hydrogels promotes angiogenesis [48]. Chitosan-coated cerium oxide nanocomposites exhibited excellent antibacterial and antifungal efficacy against foodborne pathogens [49]. Petrova et al. reported a pronounced regenerative effect of nanoceria in chitosan film on mesenchymal stem cells [50]. Chitosan–gelatin hydrogels containing ceria nanoparticles exhibited antioxidant, regenerative, and anti-inflammatory properties in diabetic wounds, inducing re-epithelialization, collagen deposition, and angiogenesis [51]. Multifunctional nanocomplexes based on nanoceria and chitosan are being actively developed for various medical purposes, where the chitosan matrix provides compatibility [52,53]. The toxicity of chitosan-coated nanoceria was studied in relation to aquatic plants. Bare nanoceria significantly inhibits duckweed growth, cause oxidative damage and lead to cell death, while chitosan or alginate coatings reduce its toxicity [54].

Thus, chitosan-coated nanoceria is not only a promising biologically active complex, but also an excellent platform for immobilization of various compounds. However, there are no or very few studies devoted to the effect of chitosan-coated nanoceria on human genes.

To sum up, coating nanoceria with maltodextrin or chitosan may be a promising option for the synthesis of new nanopharmacological preparations. Understanding the effects of surface modifiers on the efficacy and safety of cerium dioxide is required to assess the benefits and risks in medical applications. Inhalation of ceria nanoparticles in the air by personnel in the working area is one of the main routes of ceria intoxication. Human embryonic lung fibroblasts are a widely used, reliable, and sensitive model for studying the effects of various substances (including metal nanoparticles) on genes.

Here, we aimed to study the effects of maltodextrin- or chitosan-coated nanoceria on oxidative metabolism genes in human embryonic lung fibroblasts by examining (1) cell viability and mitochondrial potential, (2) intracellular ROS, (3) expression of NOX4, NRF2, NF-κB, and STAT3 proteins, (4) oxidative DNA damage/repair, and (5) cell proliferation and autophagy.

## 2. Results

### 2.1. Synthesis and Physicochemical Characterization of Nanoparticles

An electrostatically stabilized colloidal solution of nanocrystalline cerium dioxide was obtained by thermal hydrolysis of the aqueous solution of cerium(IV) ammonium hexanitrate [55]. The concentration of the aqueous cerium dioxide solution was 0.120 ± 0.001 mol/L (20.6 g/L).

Table 1 shows the particle sizes and hydrodynamic diameters of nanoparticles determined with powder X-ray diffraction (XRD) and dynamic light scattering (DLS), as well as zeta potentials.

The XRD data confirm that both bare and coated nanoceria nanoparticles contain single-phase fluorite-type cerium dioxide (PDF2 34-0394). Surface modification with polysaccharide ligands does not significantly affect the diffraction patterns. The positions of the (111), (200), (220), and (311) peaks did not change (see Supplementary Figure S1a). Calculations using the Scherrer formula resulted in particle sizes of 3–4 nm (Table 1).

The hydrodynamic diameters of nanoparticles measured in aqueous solutions with dynamic light scattering are larger than the particle sizes based on XRD data (see Supplementary Figure S1b,d). This is probably due to the aggregation of CeO_2_ nanoparticles. According to DLS, the largest hydrodynamic diameter was characteristic of chitosan-coated nano-CeO_2_ (Table 1).

The ζ-potential value for bare CeO_2_ of +38.4 ± 0.1 mV proves high stability of the aqueous solution. Polysaccharide coatings lead to a decrease in the ζ-potential values (Table 1) that indicates a change in the stabilization mechanism of these colloidal systems. The weakening of the electrostatic repulsion between coated CeO_2_ nanoparticles is compensated by the steric effect of the ligands [56,57], and therefore, coated CeO_2_ solutions are also stable.

Calculations suggest that molar ratios of ligands to cerium dioxide of 0.07:1 for maltodextrin and 0.04:1 for chitosan are required to achieve complete coating of the nanoparticles (see Supplementary Materials). Therefore, at a ligand/CeO_2_ ratio of 1:1, which represents an excess concentration of approximately one and a half orders of magnitude, we can confidently assume that nanoceria is fully coated with maltodextrin and chitosan.

### 2.2. Spectral Characterization

The electronic absorption spectra of both bare and ligand-coated nanoceria have a characteristic band in the 280–300 nm region (see Supplementary Figure S2). Thus, surface modification of CeO_2_ nanoparticles with maltodextrin or chitosan did not change the spectral characteristics.

To examine the binding affinity of the polysaccharide molecules to the surface of CeO_2_ nanoparticles, Fourier transform infrared spectroscopy with attenuated total reflectance (ATR-FTIR spectroscopy) was used (see Supplementary Figure S3). The FTIR spectrum of pure maltodextrin contains an intense broad band at about 3350 cm^−1^ (see Supplementary Figure S3a) corresponding to O–H stretching vibrations [58]. The region of 2900–2800 cm^−1^ corresponds to C–H stretching vibrations of alkyl groups (see Supplementary Figure S3a). The bands at 1642 and 1410 cm^−1^ are due to O–H bending vibrations, likely indicating interactions with adsorbed water or hydroxyl groups in the structure (see Supplementary Figure S3b). Several intense bands in the range 1200–1000 cm^−1^ (1150, 1078, and 1020 cm^−1^) (see Supplementary Figure S3b) are due to C–O and C–O–C stretching vibrations (glycosidic bonds), which is typical for polysaccharides, including maltodextrin [59].

The FTIR spectrum of the maltodextrin-coated CeO_2_ nanoparticles shows a shift and a change in the intensity of the band around 3350 cm^−1^ (see Supplementary Figure S3a). This occurs due to the interaction of the hydroxyl groups of maltodextrin with the surface of the nanoparticles and indicates the formation of hydrogen bonds or coordination interactions between the hydroxyl groups and the surface atoms of cerium. The lack of significant shifts in the bands at 1150 and 1020 cm^−1^ suggests that the uncharged ligand molecules bind to the nanoscale CeO_2_ through non-covalent interactions (see Supplementary Figure S3b) [60]. New absorption bands at 465 and 293 cm^−1^ are associated with vibrations of the Ce–O bonds in the ceria nanoparticles, proving the presence of CeO_2_ [60,61].

The FTIR spectrum of pure chitosan (see Supplementary Figure S3) exhibits a broad intense band in the region of 3400–3200 cm^−1^, due to the stretching vibrations of O–H and N–H groups [62,63,64]. The peaks at 2880 cm^−1^ and 2940 cm^−1^ correspond to the symmetric and asymmetric stretching vibrations of C–H and –CH_2_ groups, respectively [62,63,64,65]. The band at 1619 cm^−1^ is assigned to the stretching vibrations of the carbonyl group (C=O) of the acetamide fragment (amide I) [64,66]. The peak at 1510 cm^−1^ probably corresponds to the amide bands associated with deformation vibrations of the N–H and C–N bonds [62]. The band at 1413 cm^−1^ arises from deformation vibrations of the –CH_2_ groups [64]. The peaks at 1376 cm^−1^ and 1247 cm^−1^ correspond to stretching vibrations within the chitosan main chain, specifically the C–O and –C–O–C– bonds, respectively [67]. The peaks at 1152 cm^−1^ and 1066 cm^−1^ result from C–O stretching vibrations [62,63,65,66], while the peak at 1032 cm^−1^ is associated with skeletal C–O stretching vibrations [64]. The peak at 898 cm^−1^ is due to the rocking vibration of the –NH_2_ group.

Modification of CeO_2_ nanoparticles with chitosan results in changes in the spectra. The broad band near 3400–3200 cm^−1^ shifts (see Supplementary Figure S3c), indicating altered hydrogen bonding [63,64,66,67]. Bands at 2880 and 2940 cm^−1^ shift to 2854 and 2928 cm^−1^ (see Supplementary Figure S3c), showing interactions between CeO_2_ and chitosan organic groups [63]. The 1619 cm^−1^ band remains unchanged (see Supplementary Figure S3d), preserving amide groups. The 1510 cm^−1^ peak shifts to 1465 cm^−1^ (see Supplementary Figure S3d), likely due to amino group interactions with CeO_2_. The band at 1376 cm^−1^ shifts to 1317 cm^−1^, while the peaks at 1066 and 1032 cm^−1^ shift to 1040 and 990 cm^−1^ (see Supplementary Figure S3d), respectively, indicating alterations in the C–O and C–O–C vibrational modes and bonding [62,63,64,66,67]. The 898 cm^−1^ band disappears, probably due amino group interaction with CeO_2_. The newly appeared bands at 814 and 740 cm^−1^ correspond to Ce–O bond vibrations (see Supplementary Figure S3d). A shift of the cerium band from 470 to 482 cm^−1^ confirms the formation of Ce–O bonds (800–400 cm^−1^), indicating the presence of cerium oxide in the composite [60,61].

### 2.3. Cell Viability and Mitochondrial Potential

Cell viability was examined with a standardized 72 h MTT assay. The assay is based on the reduction of yellow 3-(4,5-di methyl thiazol-2-yl)-2,5-diphenyltetrazolium bromide (MTT) to purple formazan by intracellular oxidoreductases. The MTT assay is used to assess cytotoxicity (loss of viable cells) under the impact of the studied compounds. The results are presented in arbitrary units to the control values (cells incubated without the nanoparticles).

**Figure 1:**
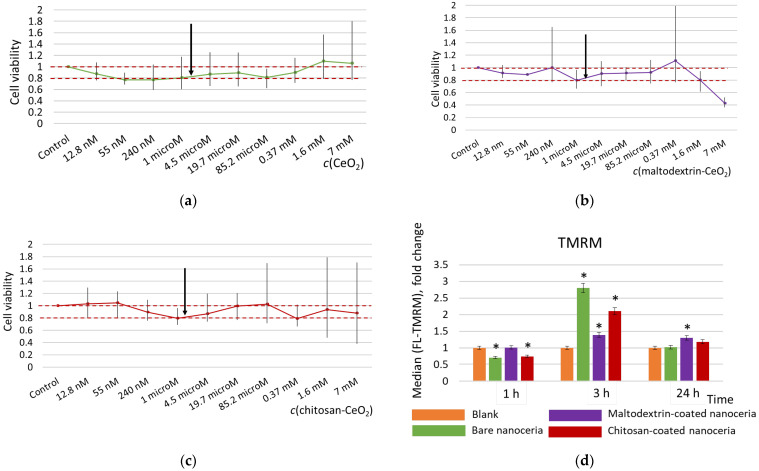
Cell viability for (**a**) bare nanoceria, (**b**) maltodextrin-coated nanoceria, and (**c**) chitosan-coated nanoceria assessed with a 72 h MTT test; the red dotted line indicates the viability limits of 80–100%, and the arrows indicate a concentration of 1.5 μmol/L selected for the further experiments. (**d**) Mitochondrial membrane potential for the cells exposed to bare, maltodextrin-, and chitosan-coated nanoceria (1.5 μmol/L); the data are presented in arbitrary units compared to the control values, and the control cells were incubated without nanoparticles. ‘*’ indicates significant differences relative to the control using the Mann–Whitney U test at a significance level of *p* = 0.05.

For all of the materials studied, cell viability plots showed a similar pattern (Figure 1a–c). Maltodextrin-coated nanoceria was more toxic at higher concentrations (>1.6 mmol/L). Chitosan coating did not affect cell viability. A concentration of 1.5 μmol/L was chosen for protein expression experiments, corresponding to the middle of the studied range and providing satisfactory viability for all three nanosubstances.

Tetramethylrhodamine, methyl ester (TMRM) is a cell-permeant, cationic red-orange, fluorescent dye that accumulates in active mitochondria with intact membrane potentials. When mitochondrial potential is lost, the fluorescence intensity decreases. When mitochondrial activity increases, fluorescence intensity increases. Compared to bare nanoceria, the polysaccharide-coated nanoceria has a milder impact on the mitochondrial membrane potential assessed with the TMRM test, especially maltodextrin-coated nanoparticles (Figure 1d). The maximum increase in the potential for all of the materials occurs within 3 h of exposure. After 24 h, the effect on the mitochondrial membrane potential for bare ceria and chitosan-coated nanoceria returns to the control values, while for maltodextrin-coated ceria it remains slightly higher than the control.

### 2.4. Visualization of Cells with Fluorescence Microscopy

**Figure 2:**
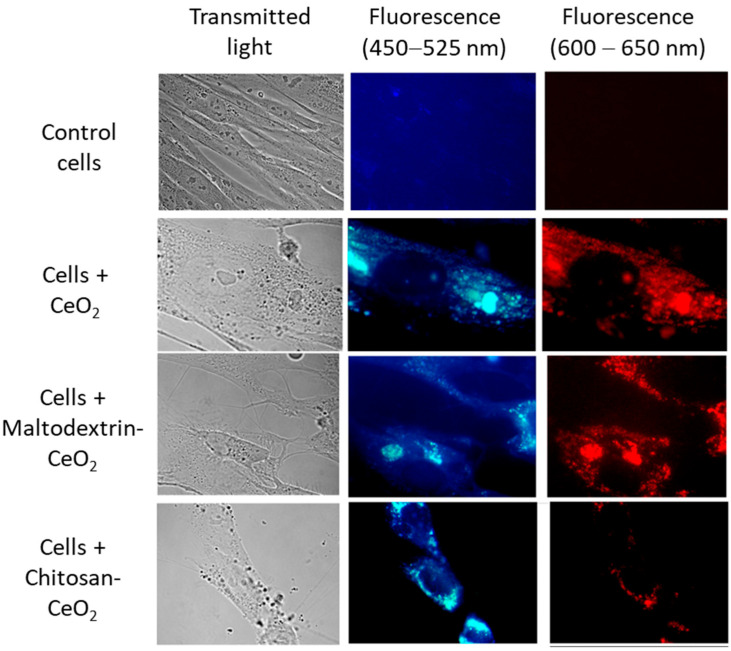
Transmitted light (**left**) and fluorescence (**middle** and **right**) images of bare nanoceria, maltodextrin-coated nanoceria, and chitosan-coated nanoceria (1.5 μmol/L) in human fetal lung fibroblasts after 3 h of exposure. Magnification, 100×; (**middle**) blue filter image (450–525 nm); and (**right**) red filter image (600–650 nm).

The intrinsic red fluorescence of nanoceria allows for the visualization of its accumulation in cells [68]. Images of control cells and cells exposed to nanoparticles (1.5 μmol/L) for 3 h are shown in Figure 2. The images indicate that bare, maltodextrin-, and chitosan-coated nanoceria nanoparticles actively enter the cells.

### 2.5. Quantitation of Intracellular Reactive Oxygen Species with Flow Cytometry

Intracellular ROS content was quantified using flow cytometry with H_2_DCFH-DA (2′,7′-dichlorodihydrofluorescein diacetate). H_2_DCFDA is a cell-permeant chemically reduced form of fluorescein. In cells, H_2_DCFDA reacts with intracellular esterases and oxidizes, converting to the highly fluorescent 2′,7′-dichlorofluorescein (DCF). Using flow cytometry, this assay allows quantitative assessment of the intracellular reactive oxygen species (primarily H_2_O_2_). An increase in DCF fluorescence indicates an increase in the level of ROS and the development of intracellular oxidative stress.

**Figure 3:**
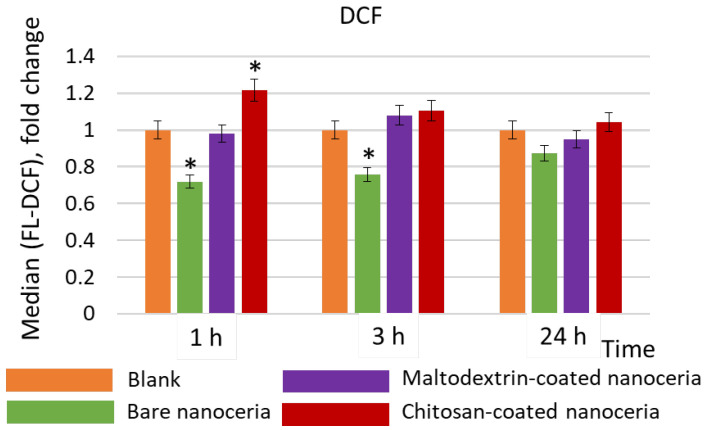
Intracellular ROS concentrations compared to the control values assessed by flow cytometry with dichlorofluorescein (DCF), a product of intracellular oxidation of 2′,7′-dichlorodihydrofluorescein diacetate; the cells were exposed to bare, maltodextrin-, and chitosan-coated nanoceria (1.5 μmol/L) within 1, 3, and 24 h. The data are presented in arbitrary units compared to the control values; the control cells were incubated without nanoparticles. ‘*’ indicates significant differences relative to the control using the Mann–Whitney U test at a significance level of *p* = 0.05.

Bare nanoceria exhibits an antioxidant effect within 1 and 3 h of exposure, while maltodextrin-coated nanoceria has no effect, and chitosan-coated nanoceria causes an increase in ROS content by ~20% within 1 h (Figure 3).

### 2.6. Genotoxicity and DNA Repair

**Figure 4:**
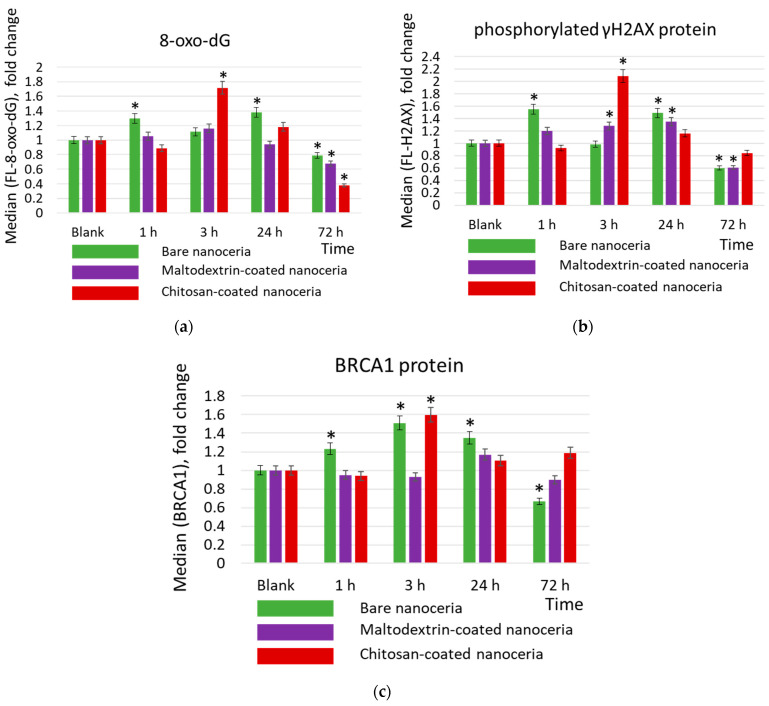
Concentrations of (**a**) 8-oxo-2′-deoxyguanosine (8-oxo-dG), (**b**) phosphorylated γH2AX, protein, and (**c**) BRCA1 protein in cells exposed to bare, maltodextrin-, and chitosan-coated nanoceria (1.5 μmol/L) for 1–72 h. The data are presented in arbitrary units compared to the control values; the control cells were incubated without nanoparticles. ‘*’ indicates significant differences relative to the control using the Mann–Whitney U test at a significance level of *p* = 0.05.

Genotoxicity was assessed by expression of 8-oxo-2′-deoxyguanosine (8-oxo-dG) and phosphorylated histone γH2AX (H2A histone family member X). 8-Oxo-dG is one of the major products of DNA oxidation, and the concentration of intracellular 8-oxo-dG is a measure of oxidative stress. Phosphorylation of γH2AX is one of the first cellular responses to DNA damage; this protein is a biomarker of double-strand breaks (Figure 4a,b).

DNA damage activates the repair system. The activity of the repair systems was assessed by BRCA1 protein (breast cancer type 1 susceptibility protein) (Figure 4c). BRCA1 protein is responsible for repairing double-strand DNA breaks and is a key DNA repair biomarker.

As for DNA oxidation, bare nanoceria exerts a ‘two-wave’ effect at 1 and 24 h. After 72 h, the concentration of 8-oxo-2′-deoxyguanosine decreases slightly below the control value (~80%) (Figure 4a). Similar dynamics are characteristic of the double-strand break marker phosphorylated histone H2AX (Figure 4b). Oxidative damage leads to the activation of the repair systems, which was assessed by the expression of the BRCA1 protein. Maximum expression is achieved within 3 h of exposure, then it decreases below the control value (Figure 4c). Maltodextrin-coated nanoceria does not cause oxidative DNA damage but leads to a decrease in 8-oxo-2′-deoxyguanosine concentration, after 72 h, below the control value (~70%) (Figure 4a). Similar dynamics are characteristic of the double-strand break marker phosphorylated histone H2AX (Figure 4b). The lack of DNA oxidation correlates with no activation of the repair systems (Figure 4c). Chitosan-coated nanoceria causes DNA damage after 3 h of exposure. After 72 h, the concentration of 8-oxo-2′-deoxyguanosine decreases below the control value (~40%) (Figure 4a). Similar dynamics are characteristic of the double-strand break marker phosphorylated histone H2AX (Figure 4b). Maximum expression of the BRCA1 protein is achieved after 3 h of exposure, then it decreases to the control value (Figure 4c).

### 2.7. ROS-Dependent and Inflammation Signaling Pathways

**Figure 5:**
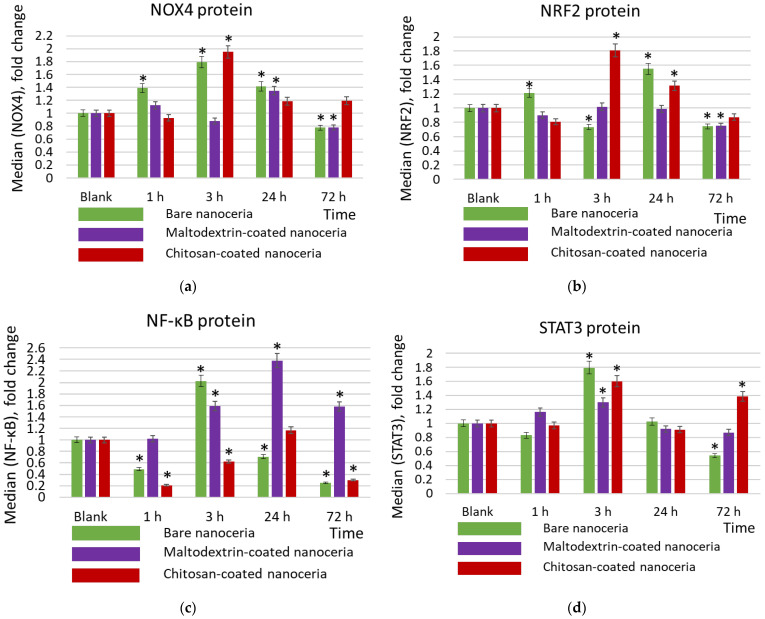
Expression of proteins (**a**) NOX4, (**b**) NRF2, (**c**) NFκB, and (**d**) STAT3 in cells exposed to bare, maltodextrin-, and chitosan-coated nanoceria (1.5 μmol/L) for 1–72 h. The data are presented in arbitrary units compared to the control values; the control cells were incubated without nanoparticles. ‘*’ indicates significant differences relative to the control using the Mann–Whitney U test at a significance level of *p* = 0.05.

The key prooxidant enzyme in cells is the membrane complex NOX4 (NADPH oxidase 4). NOX4 is a constitutive oxidase that catalyzes the formation of the superoxide radical by transferring an electron from NADPH to oxygen. Expression of NOX4 is maximal for bare and chitosan-coated nanoceria after three hours of exposure (Figure 5a). This trend correlates with the compensatory activation of the anti-inflammatory pathway NRF2 (nuclear factor E2-related factor 2) (Figure 5b). NRF2 is a transcription factor in cell cytoplasm. When activated by oxidative stress, it enters the nucleus, binds to antioxidant response elements, and activates expression of genes involved in antioxidant and anti-inflammatory responses. The dynamics of NOX4 and NRF2 expression correspond to the dynamics of oxidative DNA damage for both nanoparticles (compare Figure 4a,b and Figure 5a,b). Chitosan-coated nanoceria exerts a more pronounced impact than bare nanoceria (Figure 5a,b). Maltodextrin-coated nanoceria causes insignificant activation in the expression of NOX4 after 24 h, which decreases below the control value after 72 h (Figure 5a), but actually has no effect on the NRF2 pathway (Figure 5b).

The studied samples have different impacts on the proinflammatory NF-κB pathway (nuclear factor kappa-light-chain-enhancer of activated B cells). NF-κB is a transcription factor that is activated by various stimuli, including cytokines, bacterial and viral products, ROS, and stress factors. The NF-κB signaling pathway activates the transcription of genes involved in the inflammatory response and protection against apoptosis. Bare nanoceria activates the expression of the NF-κB protein after 3 h of exposure, while at other time points (1, 24, and 72 h) it suppresses the expression (Figure 5c). Maltodextrin-coated nanoceria has a pronounced activating impact within 3, 24, and 72 h of exposure (Figure 5c). Chitosan-coated nanoceria inhibits NFκB expression for 1 h, then by 24 h, the activity of the pathway is restored, and after 72 h it decreases below the control level (Figure 5c).

STAT3 (signal transducer and activator of transcription 3) mediates the cell’s response to signals through interleukin and growth factor receptors. This signaling pathway is involved in inflammation and carcinogenesis. For the STAT3 pathway, the activating impact of all nanosubstances is maximal for 3 h of exposure, with maltodextrin-coated nanoceria having the mildest effect (Figure 5d). It is seen that the dynamics of STAT3 changes correspond to the dynamics of NOX4 changes.

### 2.8. Autophagy and Proliferation

**Figure 6:**
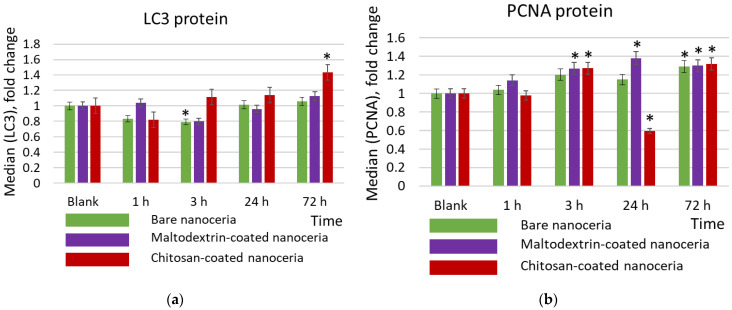
Expression of (**a**) the proliferation marker PCNA protein, (**b**) the autophagy marker LC3, and (**c**) BCL2 protein in cells exposed to bare, maltodextrin-, and chitosan-coated nanoceria (1.5 μmol/L) for 1–72 h. The data are presented in arbitrary units compared to the control values; the control cells were incubated without nanoparticles. ‘*’ indicates significant differences relative to control using the Mann–Whitney U test at a significance level of *p* = 0.05.

Autophagy is a process in which internal cell components are degraded in lysosomes or vacuoles. Microtubule-associated protein 1A/1B-light chain 3 (LC3) is a reliable biomarker for autophagy and autophagy-related processes [69]. As for LC3, chitosan-coated nanoceria causes an activating effect after 72 h of exposure, in contrast to bare and maltodextrin-coated nanoceria, which are characterized by a weak suppressive effect after 3 h of exposure, and then the LC3 content does not differ from the control value (Figure 6a).

PCNA nuclear protein is a cofactor for DNA polymerase delta, and its expression is increased when DNA replication occurs. Based on the expression of the proliferation biomarker PCNA (proliferating cell nuclear antigen), bare, maltodextrin-, and chitosan-coated nanoceria give comparable proliferative effects within 1, 3, and 72 h of exposure. However, chitosan-coated nanoceria suppresses PCNA expression at 24 h. (Figure 6b).

**Figure 7:**
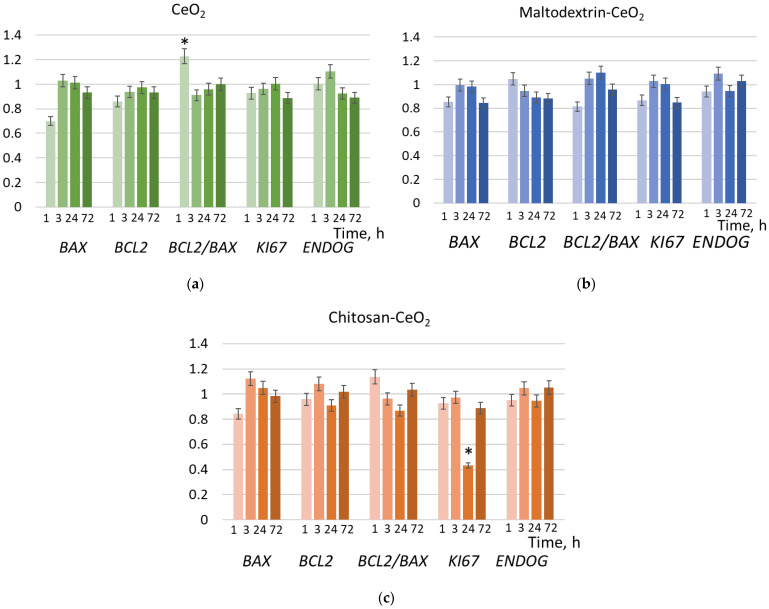
Expression of the genes, compared to the control values, in cells exposed to (**a**) bare, (**b**) maltodextrin-coated, and (**c**) chitosan-coated nanoceria (1.5 μmol/L) for 1, 3, 24, and 72 h; ‘*’ indicates significant differences relative to control using the Mann–Whitney U test (*p* = 0.05). Control cells were incubated without nanoparticles.

To prove the suppressing effect of chitosan-coated nanoparticles on proliferation within 24 h, we studied the expression of genes involved in proliferation by the real-time quantitative reverse transcription polymerase chain reaction method (qRT-PCR). These are the proapoptotic gene *BAX*, the anti-apoptotic gene *BCL2,* and their ratio, *BCL2/BAX*, as well as *Ki-67* (a gene encoding the protein, which is a proliferation marker) and *ENDOG* (mitochondrial endonuclease G) (Figure 7). From this data, it follows that incubation of nanoparticles with cells has a minimal effect on apoptosis. However, indeed, at 24 h, chitosan-coated CeO_2_ causes a significant decrease in the expression of the *Ki-67* gene by more than two times (Figure 7c).

## 3. Discussion

Nanoceria is considered a promising nanopharmaceutical due to its regenerative and proliferative impacts on normal cells and cytotoxic impacts on cancer cells. In medical applications of inorganic nanomaterials, surface modifiers of various chemical structures are actively used for stabilization of colloidal solutions, facilitation of cell penetration, and improvement of targeted efficiency. The development of multifaceted nanoparticles lays the foundation for novel platforms for targeted impacts on cells and tissues. Here, we have studied two polysaccharide surface modifiers, maltodextrin and chitosan, compared with bare nanoceria.

The main results for maltodextrin-coated nanoceria can be summarized as follows: (1) maltodextrin-coated nanoceria remains safe over a broad concentration range and has minimal activating impact on mitochondrial potential within 24 h; (2) the nanoparticles penetrate cells actively within 3 h; (3) the maltodextrin-coated nanoparticles do not affect intracellular ROS balance and do not cause DNA oxidation and activation of DNA repair systems; (4) exposure of the nanoparticles to cells leads to slight activation of NOX4 within 24 h and strong activation of proinflammatory NF-κB pathway within 24 and 72 h; and (5) the maltodextrin coating keeps the proliferative properties of bare nanoceria without activating autophagy.

The main results for chitosan-coated nanoceria can be summarized as follows: (1) chitosan-coated nanoceria remains safe over a wide concentration range and has activating impact on mitochondrial potential within 3 h; (2) nanoparticles penetrate cells actively within 3 h; (3) unlike bare nanoceria, chitosan-coated nanoparticles induce short-term intracellular oxidative stress within 1 h of exposure, which after 3 h leads to DNA oxidation, double-strand breaks, and activation of the DNA repair systems; (4) activation of NOX4 and STAT3 occurs within 3 h along with the compensatory anti-inflammatory NRF2 pathway, and marked inhibition of the proinflammatory NF-κB pathway within 1, 3, and 72 h; and (5) cell proliferation is inhibited after 24 h of exposure, but autophagy is activated within 72 h.

**Figure 8:**
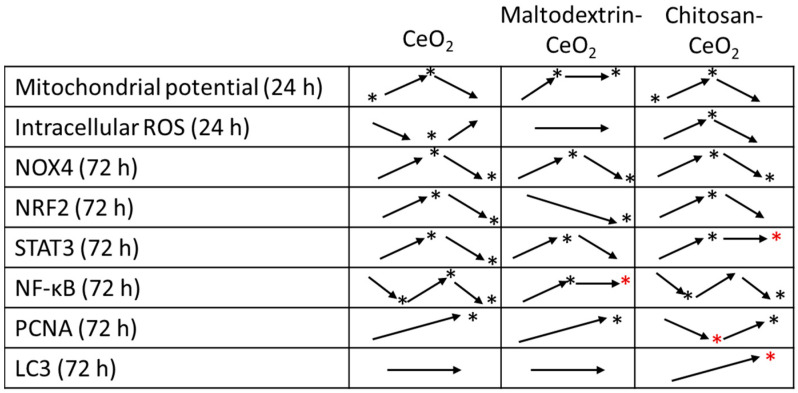
The dynamics of changes in the studied parameters (the time intervals are indicated in the first column) for nanoceria samples; ‘*’ marks significant changes compared to the control.

The key trends are illustrated in Figure 8. Red asterisks mark the most interesting differences with bare nanoceria.

To sum up, maltodextrin-coated nanoceria exerts milder influence on oxidative metabolism (NOX4, NRF2, and STAT3 pathways) compared to bare nanoceria. There is no DNA damage and no intracellular oxidative disbalance. Impacts of chitosan-coated nanoceria are similar to those of bare nanoceria (mitochondrial potential, DNA oxidation, and activation of NOX4, NRF2, and STAT3). However, there is a specific activation of autophagy and inhibition of proliferation within 24 h. Interesting differences are found for the proinflammatory NF-κB pathway. Bare nanoceria results in NF-κB activation within 3 h of exposure followed by its inhibition below the control values within 24–72 h. Chitosan-coated nanoceria has similar time dynamics but with more pronounced effects, and the increase in NF-κB activity at 24 h does not exceed the control values. Maltodextrin-coated nanoceria, on the contrary, causes activation of this pathway at 3, 24, and 72 h.

Below, we discuss the most interesting findings, namely, the activation of autophagy and the STAT3 pathway as well as inhibition of PCNA expression by chitosan-coated nanoceria and the effect of maltodextrin-coated nanoceria on the NF-κB pathway.

Autophagy is a complex process of recycling organelles and macromolecules that accompanies the life of any normal cell under normal conditions. This process is important in protein quality control to promote cell survival. The mechanisms of autophagy are complex and not fully understood. For example, autophagy can directly control the transcription factor NF-κB [70]. Most studies on autophagy regulation are devoted to complex nanoparticles containing chitosan [71,72,73,74], and it is difficult to isolate its contribution to autophagy. However, there are several works where pure chitosan is studied. Yang et al. reported that culturing mesenchymal stem cells on a chitosan substrate resulted in enhanced calcium-dependent autophagy [75]. In senescent stem cells, chitosan activates the mTOR pathway, which is a key signaling pathway that regulates cell growth, proliferation, metabolism, and autophagy [76]. There is a close association between autophagy and the apoptosis of cancer cells, which use autophagy for survival. Wang et al. studied the effect of chitosan nanoparticles on autophagy in tumor cells. They demonstrated that these nanoparticles at non-toxic concentrations in the range of 10 to 100 μg/mL triggered ROS-mediated autophagy [77]. Chitosan oligosaccharide exhibited autophagy-activating and anti-inflammatory effects in a chondrocyte inflammation model [78]. Chitosan oligosaccharide activated renal autophagy in prediabetic rats [79]. Chitosan oligosaccharide reduced inflammation in the *substantia nigra* (a model of Parkinson’s disease) and activated autophagy, ultimately alleviating the course of Parkinson’s disease in mice [80]. Thus, our studies are in agreement with other scientific data confirming that including chitosan in a complex with nanoceria leads to the activation of autophagy. In our study, chitosan-coated nanoceria caused short-term oxidative stress in cells. It is possible that this is one of the reasons for the activation of autophagy.

As for proliferation, carnosine-coated nanoceria was similar to bare nanoceria, maintaining proliferative properties at 1, 3, and 72 h of exposure, but inhibited PCNA expression at 24 h of exposure. Chitosan nanoparticles promoted increased proliferation (including increased PCNA expression) in a rat model of hepatotoxicity [81]. Howling et al. showed increased proliferation of human fibroblasts by chitosan and suggested that chitosan interacted with growth factors in the serum present in the culture medium [82]. Chitosan exerted a proliferative effect on human skin fibroblasts [83], and stimulated gingival fibroblast cell proliferation via the ERK1/2 pathway [84]. Cerium oxide hydrogels with chitosan/gelatin promote fibroblast migration in mice and diabetic wound healing [51]. Cryogels of nanoceria, chitosan, and tannic acid stimulated fibroblast adhesion and proliferation [47]. Thus, the proliferative activity of chitosan-coated nanoceria is consistent with the literature data. However, there are several studies demonstrating the inhibitory effect of chitosan on rabbit tendon fibroblasts [85,86]. Proliferation is an extremely complex process with a multilayered regulatory mechanism finely tuned to DNA damage. Further studies are obviously needed to elucidate the basis for the “oscillating” effect of chitosan-coated nanoceria on proliferation.

Unlike bare nanoceria, which exhibits a pronounced inhibitory effect on STAT3 protein expression, chitosan-coated nanoceria induced activation of the STAT3 pathway within 72 h. STAT3 mediates cell responses to signals from interleukins and growth factor receptors and plays an important role in cell survival, proliferation, differentiation, angiogenesis, and immune responses [87]. Chang et al. cultured cancer stem cells on chitosan membranes and proved that chitosan activated the Wnt-STAT3 signaling in CD44– hepatocellular carcinoma cells [88]. Carboxymethyl chitosan, which is a soluble chitosan derivative, attenuated inflammation in rat chondrocytes by significantly decreasing iNOS (inducible nitric oxide synthase) expression, upregulating the anti-inflammatory cytokine IL-10, and increasing the expression of JAK1 (Janus kinase 1), STAT3, and SOCS3 (suppressor of cytokine signaling 3). The authors conclude that carboxymethyl chitosan activates the JAK/STAT/SOCS signaling pathway [89]. Pan et al. showed that chitosan oligosaccharide capsules in an in vivo rat model activate the JAK2-STAT3 signaling pathway [90]. Thus, our data on the possible contribution of chitosan to STAT3 activation are supported by literature data.

Maltodextrin coating leads to activation of NF-κB expression within 72 h. The transcription factor NF-κB is located in the cytoplasm and upon activation moves to the nucleus. There are two pathways for NF-κB activation, which require complex molecular interactions with adaptor proteins and phosphorylation and ubiquitinase enzymes [91]. NF-κB is activated by endotoxins, inflammatory stimuli, carcinogens, pathogens, nicotine, and tumor promoters. NF-κB controls the expression of 400 different genes, including various enzymes, cytokines, viral proteins, and regulatory molecules involved in the cell cycle. This pathway is associated with a variety of diseases, including inflammatory diseases, autoimmune diseases, cancer, and diabetes [92]. Dysregulated NF-κB signaling results in unregulated cell proliferation, viability, motility, and invasion, thereby promoting tumor development. The NF-κB pathway is one of the most frequently disrupted signaling cascades in human cancer, playing a key role in cell growth, survival, and resistance to therapy [93,94].

There are no anti-inflammatory effects of maltodextrin reported in the literature. On the contrary, maltodextrin feeding was used as a control in the study of the inflammatory effect of ethanol [95,96]. In our study, modification with maltodextrin reduced the prooxidant capacity of cerium dioxide towards hydrogen peroxide by six times, which is consistent with the “softening” effect of maltodextrin on the properties of nanoceria [97]. In addition, maltodextrin coating increases the superoxide dismutase-like activity of nanoceria and the antioxidant capacity towards alkyl peroxyl radicals [60]. However, maltodextrin coating caused an increase in mitochondrial membrane potential lasting up to 24 h. This is consistent with the data of So et al., who cultured the human colorectal cancer cell line HCT116 with resistance maltodextrin (FIbersol-2) and found that Fibersol-2 significantly suppressed tumor growth of HCT116 cells by inducing apoptosis, inducing mitochondrial ROS and Bax-dependent cleavage of caspase 3 and 9, and phosphorylation of Akt/mTOR [98]. Indeed, membrane potential is a powerful regulator of the mitochondrial generation of reactive oxygen species, which may underlie the connection with ROS-dependent signaling pathways [99]. The mechanism of the “crosstalk” between mitochondria and the NF-κB signaling pathway is extremely complex, but mitochondrial dynamics are associated with NF-κB activity during neuronal stress adaptation [100]. Further research is needed to study this phenomenon.

## 4. Materials and Methods

### 4.1. Synthesis of Bare and Ligand-Coated CeO_2_ Nanoparticles

The electrostatically stabilized colloidal CeO_2_ solution was synthesized through the thermal hydrolysis of ammonium cerium(IV) nitrate (#215473, Sigma, St. Louis, MO, USA) [55]. An aqueous solution of (NH_4_)_2_Ce(NO_3_)_6_ (100 g/L) was maintained in a drying oven at 95 °C for 24 h. The precipitate formed was separated by centrifugation, washed three times with isopropanol, and then redispersed in deionized water. This suspension was boiled for 1 h with continuous stirring to ensure complete evaporation of isopropanol (boiling point 82.6 °C). Solutions of maltodextrin (#419672, *n* = 4–7, Sigma, St. Louis, MO, USA) and chitosan (#448869, Sigma, St. Louis, MO, USA) were prepared using deionized water. The ligand-coated CeO_2_ nanoparticles were produced by slowly adding the CeO_2_ solution dropwise into the ligand solutions to achieve a 1:1 molar ratio while stirring continuously. After the complete addition of CeO_2_, stirring continued for an additional 30 min, allowing ligand molecules to adsorb onto the surface of the CeO_2_ nanoparticles.

### 4.2. Materials Characterization

The concentration of the CeO_2_ solution was measured using a gravimetric method. Portions of the CeO_2_ solution were placed into crucibles that had been pre-weighed to a constant mass and then evaporated in a muffle furnace, followed by heating at 900 °C. The samples were maintained at this temperature for 4 h.

X-ray diffraction analysis of the dried samples was conducted with a Bruker D8 Advance diffractometer (Bruker, Billerica, MA, USA) employing Cu*K*α radiation in a θ–2θ configuration over an angular range of 3–120° 2θ, with step sizes between 0.01° and 0.02° 2θ and a minimum signal accumulation time of 0.3 s per point. Diffraction peaks were identified using the ICDD PDF2 database. Particle size was determined by applying the Scherrer equation.

The average hydrodynamic diameter of the CeO_2_ nanoparticles was assessed through dynamic light scattering (DLS) using a Photocor Compact-Z analyzer (Photocor, Moscow, Russia) equipped with a diode laser (λ = 650 nm, 25 mW). Measurements were taken at a 90° scattering angle and ambient temperature. Zeta potential measurements were performed with a Nano ZS Zetasizer (Malvern Panalytical, Malvern, Worcestershire, UK) following the ISO/TR 19997:2018 standard [101]. For both hydrodynamic size and zeta potential analyses, aqueous solutions of CeO_2_ at a concentration of 1.5 mM in 2 mL volumes were used.

UV-visible absorption spectra were recorded at room temperature using a Cary 4000 UV-Vis spectrophotometer (Agilent Technologies, Santa Clara, CA, USA) with 1.0 cm path length quartz cuvettes.

To evaluate the binding of ligand molecules on the surface of CeO_2_ nanoparticles, attenuated total reflectance-Fourier transform infrared spectroscopy (ATR-FTIR) was employed. The FTIR spectra were obtained using a VERTEX 70 Fourier spectrometer (Bruker, Billerica, MA, USA) equipped with a diamond crystal on a GladiATR™ ATR accessory (PIKE Technologies, Madison, WI, USA). Spectral data were collected over the range of 4000 to 150 cm^−1^, with 64 scans for both sample and background, at a resolution of 2 cm^−1^ and a crystal temperature maintained at 50 °C. For solution measurements, up to 5 μL of the sample was placed on the ATR plate and allowed to dry completely for 4 to 5 min prior to analysis.

### 4.3. Cell Culture

Human embryonic lung fibroblasts (the 4th passage) were provided by the Research Centre for Medical Genetics (Moscow, Russia). Cells were seeded in Dulbecco’s modified Eagle’s medium (PanEco, Moscow, Russia) with 10% fetal calf serum (PAA, Vienna, Austria), 50 U/mL penicillin, 50 μg/mL streptomycin, and 10 μg/mL gentamicin. The cell concentration was 1.7 × 10^4^ cells/mL. The cells were cultured overnight at 37 °C for 24 h. Next, nanoparticles (bare nanoceria, maltodextrin-coated nanoceria, and chitosan-coated nanoceria) were added. The exposure intervals were 1, 3, 24, and 72 h. During incubation, the culture remained subconfluent, which ensured continuous cell division.

### 4.4. Cell Viability and Mitochondrial Membrane Potential

Cell viability was assessed using a 72-h MTT assay (3′-(4,5-dimethylthiazol-2-yl)-2,5-diphenyl tetrazolium bromide assay). Fluorescence was measured at 550 nm using an EnSpire plate reader (EnSpire Equipment, Turku, Finland). Cells were exposed to nanoparticles for 72 h. In control experiments, cells were incubated without nanoparticles.

Mitochondrial membrane potential was assessed with tetramethylrhodamine methyl ester (TMRM) (Thermo Fisher, Waltham, MA, USA) as described elsewhere [102].

### 4.5. Visualization with Fluorescence Microscopy

Fluorescence images were obtained with an AxioImagerA2 microscope (Carl Zeiss, Oberkochen, Germany). Approximately 500,000 cells were seeded in slide-bottom flasks. The medium was removed, and the cells were washed with phosphate-buffered saline (PBS). Next, dichloro-dihydrofluorescein diacetate (a stock solution 2 mg/mL diluted with PBS 1:200) was added. The cells were incubated for 15 min, washed with PBS, and at least 100 fields of view were analyzed. The signal acquisition time was 6–10 s. Images were obtained in transmitted light and fluorescence modes with a blue filter (bandwidth 450–525 nm) and a red filter (bandwidth 600–650 nm). The microscope software (ZEN 3.10) was used for image processing.

### 4.6. Flow Cytometry Analysis

Protein expression and intracellular ROS quantitation in cell suspensions were performed with flow cytometry. For ROS quantitation, samples were incubated with 10 μM H2DCFH-DA in PBS (Molecular Probes/Invitrogen, Carlsbad, CA, USA) for 15 min in the dark, washed with PBS, resuspended in PBS, and analyzed in the FITC channel (CytoFlex S, Beckman Coulter, Brea, CA, USA).

For protein quantitation, cells were washed with Versene solution (Thermo Fisher Scientific, Waltham, MA, USA), treated with 0.25% trypsin (Paneko, Moscow, Russia), washed with the medium, suspended in PBS (pH 7.4) (Paneko, Moscow, Russia), fixed with paraformaldehyde (PFA, Sigma-Aldrich, Saint Louis, MO, USA) at 37 °C for 10 min, washed three times with 0.5% BSA-PBS, treated with 0.1% Triton X-100 in PBS for 15 min at 20 °C or 90% methanol at 4 °C, and washed three times with 0.5% BSA-PBS. The cells were stained with conjugated antibodies (1 μg/mL) for 2 h at room temperature, washed with PBS and analyzed with a flow cytometer (Cy-toFlex S, Beckman Coulter, Brea, CA, USA).

We used primary antibodies DyLight488-γH2AX (pSer139) (nb100-78356G, NovusBio, Centennial, CO, USA), FITC-NRF2, (bs1074r-fitc, Bioss Antibodies Inc. Woburn, MA, USA), FITC-BRCA1 (Nb100-598F, NovusBio, Centennial, CO, USA), PE-8-oxo-dG (sc-393871 PE, Santa Cruz Biotechnology, Dallas, TX, USA), CY5.5-NOX4 (bs-1091r-cy5-5, Bioss Antibodies Inc. Woburn, MA, USA), A350-BCL2 (bs-15533r-a350, Bioss Antibodies Inc. Woburn, MA, USA), NFKB (bs-0465r-cy7, Bioss Antibodies Inc. Woburn, MA, USA), LC3 (NB100-2220 NovusBio, Centennial, CO, USA), PCNA (ab2426, Abcam plc, Cambridge, UK), and secondary anti-rabbit IgG-FITC (sc-2359, Santa Cruz Biotechnology, Dallas, TX, USA).

### 4.7. Real-Time Quantitative Reverse Transcription Polymerase Chain Reaction

Total mRNA was isolated with the RNeasy Mini Kit (Qiagen, Hilden, Germany), treated with DNAse I, and then reverse transcribed by the Reverse Transcriptase kit (Sileks, Moscow, Russia). The qRT-PCR method (real-time quantitative reverse transcription polymerase chain reaction) with SYBR Green PCR Master Mix (Applied Biosystems, Foster City, CA, USA) was used for obtaining expression profiles. The mRNA was quantified using StepOnePlus (Applied Biosystems) with TBP as a reference gene. The following primers were used (Sintol, Moscow, Russia): *BAX* (F: CCCGAGAGGTCTTTTTCCGAG, R: CCAGCCCATGATGGTTCTGAT); *BCL2* (F: TTTGGAAATCCGACCACTAA; R: AAAGAAATGCAAGTGAATGA); *Ki-67* (F: ACGCCTGGTTACTATCAAAAGG; R: CAGACCCATTTACTTGTGTTGGA); *ENDOG* (F: GCAGCTACCAAAACGTCTATGT; R: CACCTTGAAGAAGTGTGTGGG); and *TBP* (reference gene) (F: GCCCGAAACGCCGAATAT, R: CCGTGGTTCGTGGCTCTCT).

### 4.8. Statistics

All experiments were performed in triplicate. The mean and standard deviation were calculated. The nonparametric Mann–Whitney U test was used to assess the significance of differences (*p* = 0.05). Statistically significant differences were calculated relative to control. In calculations, StatPlus2007 Pro v4.9.2 software (AnalystSoft Inc., Walnut, CA, USA) was used.

## 5. Conclusions

Using the in vitro model of human embryonic lung fibroblasts, we have studied the effects of maltodextrin- and chitosan-coated nanoceria on cell survival, cellular accumulation, intracellular ROS balance, oxidative DNA damage, double-strand breaks, activation of the DNA repair system, expression of proteins of the ROS-dependent signaling pathways NOX4, NRF2, NF-κB, and STAT3, expression of protein markers of proliferation, and autophagy. Polysaccharide-coated nanoceria was not toxic for the cells over a wide range of concentrations. Within 3 h, bare and polysaccharide-coated nanoceria nanoparticles are effectively internalized into cells. Maltodextrin coating mitigates the oxidative impacts of bare nanoceria—there were no changes in intracellular ROS level, no oxidative DNA damage, and no activation of repair systems with weak activation of the NOX4 pathway. Like bare nanoceria, maltodextrin-coated nanoparticles exert the proliferative effect and do not activate autophagy. However, maltodextrin-coated nanoparticles have an impact on mitochondrial potential and activate the NF-κB pathway, which may be interrelated processes. Unlike maltodextrin, chitosan-coated nanoceria causes short-term oxidative stress in cells, oxidative DNA damage, double-strand breaks, activation of repair systems, and activation of NOX4, STAT3, and NRF2. Distinctive features of chitosan-coated nanoceria involve a pronounced inhibitory effect on the proinflammatory NF-κB pathway within 1, 3, and 72 h, inhibition of proliferation within 24 h, and activation of autophagy within 72 h. These results would be useful in the development of new pharmacological drugs based on nanoparticles stabilized by polysaccharides and also contribute to the understanding of the biochemical properties of nanoceria as a regulator of ROS-dependent processes in cells. Further studies in other cell cultures and with longer exposure times are required to gain a deeper understanding of this issue.

## Figures and Tables

**Table 1:** Physicochemical characteristics of bare and polysaccharide-coated CeO_2_ nanoparticles.

Sample	Particle Size, nm (Powder X-Ray Diffraction)	Hydrodynamic Diameter, nm (Dynamic Light Scattering)	ζ, mV
Bare CeO_2_	3.0 ± 0.2	13.0 ± 0.3	38.4 ± 0.1
Maltodextrin-coated CeO_2_	2.5 ± 0.2	18.2 ± 0.2	15.2 ± 0.3
Chitosan-coated CeO_2_	4.0 ± 0.2	39 ± 3	25.1 ± 0.2

## Data Availability

The original contributions presented in the study are included in the article/Supplementary Materials. Further inquiries can be directed to the corresponding author.

## Supplementary Materials

The following supporting information can be downloaded at https://www.mdpi.com/article/10.3390/molecules30153078/s1: Estimation of ligand/nanoceria molar ratio; Figure S1. (a) X-ray diffraction patterns of the dried sols of bare and ligand-coated CeO_2_ and (b–d) hydrodynamic diameter distributions for CeO_2_ particles in aqueous sols; Figure S2. UV-vis absorption spectra of bare and ligand-coated CeO2 sols; Figure S3. Fourier-transform infrared (FTIR) spectra with attenuated total reflection (ATR) for bare CeO_2_, maltodextrin, and maltodextrin-coated CeO_2_ nanoparticles in the range of (a) 4000–2000 cm^−1^ and (b) 2000–200 cm^−1^; FTIR spectra with ATR for bare CeO_2_, chitosan, and chitosan-coated CeO_2_ nanoparticles in the range of (c) 3500–2000 cm^−1^ and (d) 1700–400 cm^−1^. References [68,103] are cited in the Supplementary Materials.

## Abbreviations

The following abbreviations are used in this manuscript:

BRCA1	Breast cancer type 1 susceptibility protein
DCF	2′,7′-dichlorofluorescein
DLS	Dynamic light scattering
FTIR-ATR	Fourier transform infrared spectroscopy with attenuated total reflectance
γH2AX	H2A histone family member X
H_2_DCFH-DA	2′,7′-dichlorodihydrofluorescein diacetate
iNOS	Inducible nitric oxide synthase
JAK	Janus kinase
LC3	1A/1B-light chain 3
MTT	3-(4,5-Di methyl thiazol-2-yl)-2,5-diphenyltetrazolium bromide
NF-κB	Nuclear factor kappa-light-chain-enhancer of activated B cells
NOX4	NADPH oxidase 4
NRF2	Nuclear factor erythroid 2-related factor 2
8-oxo-dG	8-oxo-2′-deoxyguanosine
PBS	Phosphate-buffered saline
PCNA	Proliferating cell nuclear antigen
qRT-PCR	Real-time quantitative reverse transcription polymerase chain reaction
ROS	Reactive oxygen species
SOCS3	Suppressor of cytokine signaling 3
STAT3	Signal transducer and activator of transcription 3
TMRM	Tetramethylrhodamine, methyl ester
XRD	X-ray diffraction
