# Supplementary material for: Nanoceria Coated with Maltodextrin or Chitosan: Effects on Key Genes of Oxidative Metabolism, Proliferation, and Autophagy in Human Embryonic Lung Fibroblasts
Source: Molecules. 2025 Jul 23;30(15):3078. doi: 10.3390/molecules30153078 (PMC12348771; doi:10.3390/molecules30153078)
Supplement: Supplementary file 1 [file molecules-30-03078-s001.zip › molecules-3690867-supplementary.pdf]

molecules

MDPI

Supplementary

Nanoceria Coated with Maltodextrin or Chitosan: Effects of on Key Genes of Oxidative Metabolism, Proliferation and Autophagy in Human Embryonic Lung Fibroblasts

Elena V. Proskurnina, Madina M. Sozarukova, Elizaveta S. Ershova, Ekaterina A. Savinova, Larisa V. Kameneva, Natalia N. Veiko, Vladimir P. Saprykin, Khamzat K. Vyshegurov, Vladimir K. Ivanov, Svetlana V. Kostyuk

Estimation of ligand:nanoceria molar ratio

The molar ratio of ligand to CeO_2 required to fully coat the CeO_2 nanoparticles was estimated based on their particle geometry, assuming a rigid sphere model. This calculation yields an approximate value. The molar ratio was determined using the following formulas [1,2]:

$$maltodextrin{:}\,CeO_2 = \frac{3 \cdot a^3}{TPSA \cdot D_{XRD}} = \frac{3 \cdot 5.410^3}{269 \cdot 25} = 0.07{:}\,1,$$

$$chitosan{:}\,CeO_2 = \frac{3 \cdot a^3}{TPSA \cdot D_{XRD}} = \frac{3 \cdot 5.410^3}{286 \cdot 40} = 0.04{:}\,1,$$

where *a* – unit cell parameter of CeO_2 (Å), D_{XRD} – diameter of CeO_2 nanoparticles (Å), according to X-ray diffractometry data, TPSA – topological polar surface area of ligand (Å^2) [https://pubchem.ncbi.nlm.nih.gov/#query=maltodextrin], [https://pubchem.ncbi.nlm.nih.gov/#query=chitosan].

The results indicate that at the molar ratios 1:1 ligand:CeO_2 are presented in this study, the surface of the CeO_2 nanoparticles is completely covered with the ligands.

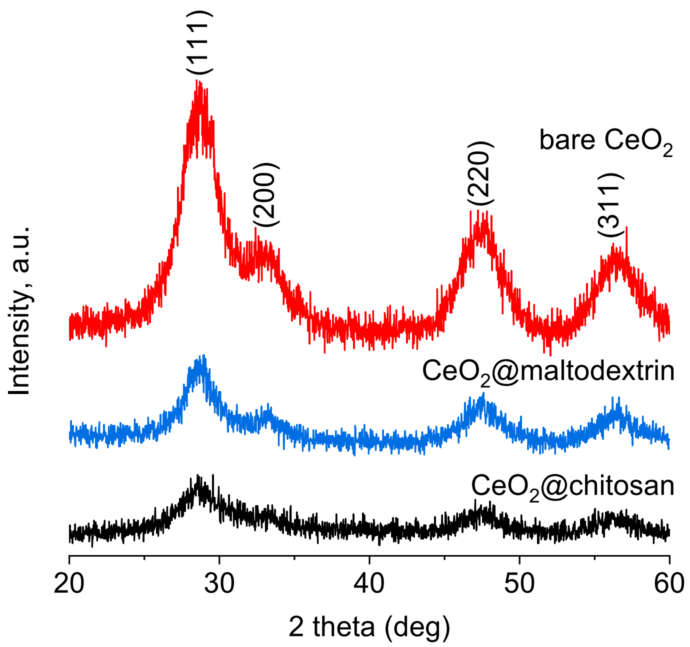

(**a**)

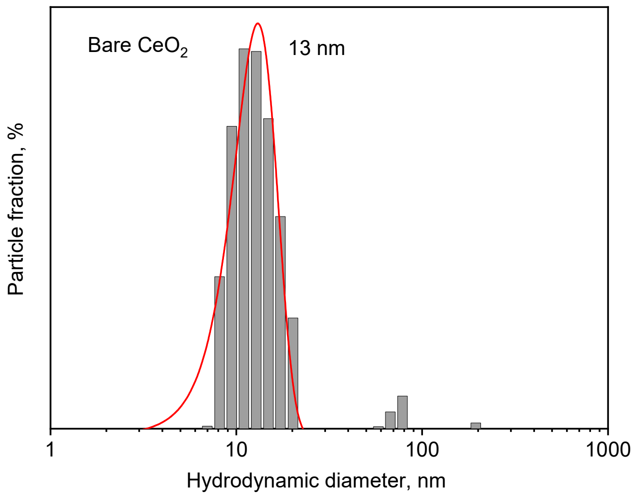

(**b**)

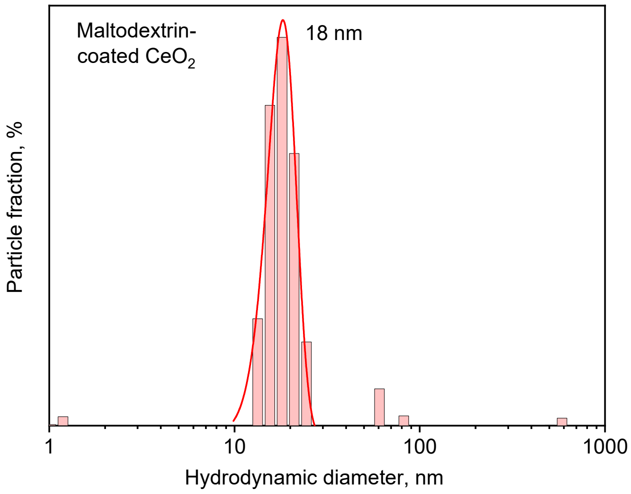

(**c**)

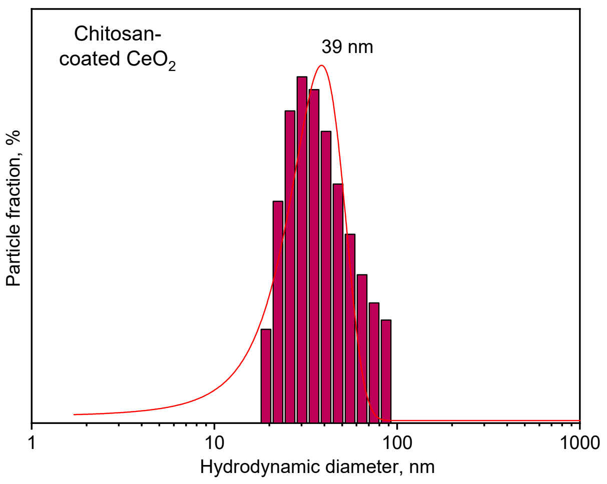

(**d**)

Figure S1. (**a**) X-ray diffraction patterns of the dried sols of bare and ligand-coated CeO_2, (**b**)–(**d**) hydrodynamic diameters distributions for CeO_2 particles in aqueous sols.

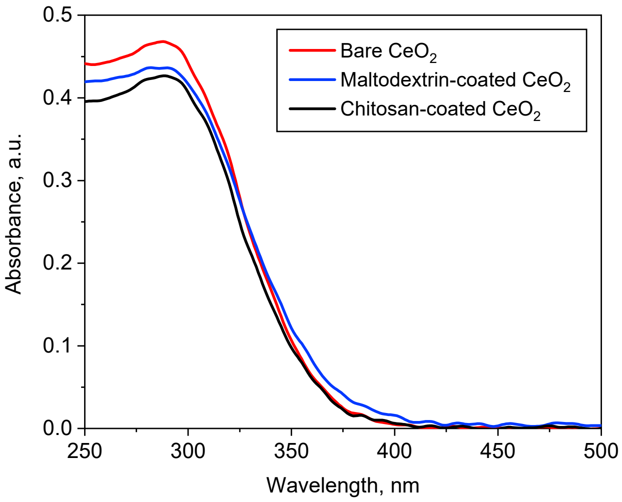

Figure S2. UV-Vis absorption spectra of bare and ligand-coated CeO_2 sols.

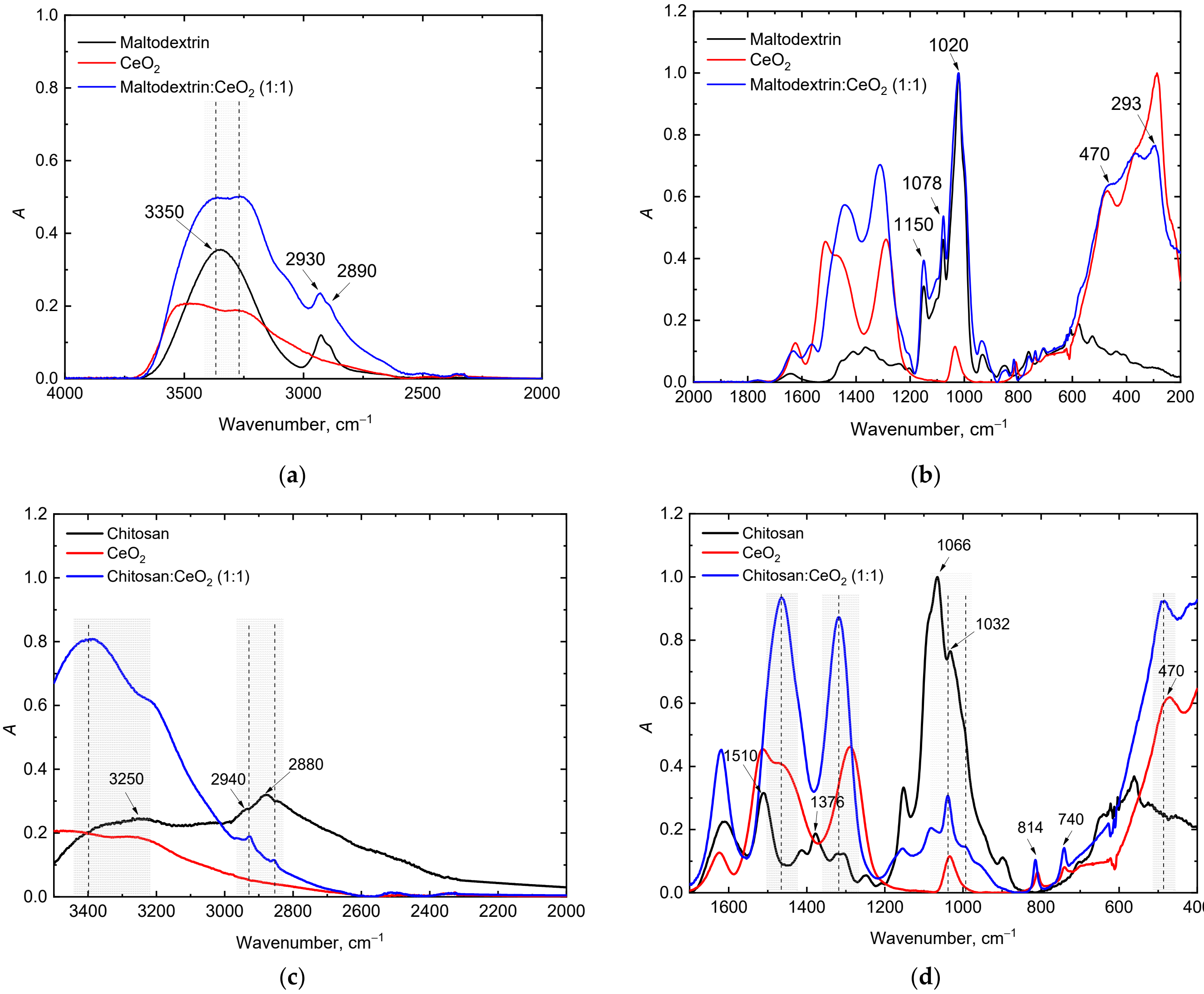

Figure S3. Fourier-transform infrared (FTIR) spectra with attenuated total reflection (ATR) for bare CeO_2, maltodextrin and maltodextrin-coated CeO_2 nanoparticles in the range of (**a**) 4000–2000 cm^{-1} (**b**) 2000–200 cm^{-1}; FTIR spectra with ATR for bare CeO_2, chitosan and chitosan-coated CeO_2 nanoparticles in the range of: (**c**) 3500–2000 cm^{-1} (**d**) 1700–400 cm^{-1}.

References

1. Filippova, A.D.; Baranchikov, A.E.; Teplonogova, M.A.; Savintseva, I.V.; Popov, A.L.; Ivanov, V.K. Ligand-to-Metal Ratio Governs Radical-Scavenging Ability of Malate-Stabilised Ceria Nanoparticles. *Nanomaterials* **2024**, *14*, 1908.
2. Proskurnina, E.V.; Sozarukova, M.M.; Ershova, E.S.; Savinova, E.A.; Kameneva, L.V.; Veiko, N.N.; Teplonogova, M.A.; Saprykin, V.P.; Ivanov, V.K.; Kostyuk, S.V. Lipid Coating Modulates Effects of Nanoceria on Oxidative Metabolism in Human Embryonic Lung Fibroblasts: A Case of Cardiolipin. *Biomolecules* **2025**, *15*, 53.
